# Clinical Significance of Combined Density and Deep-Learning-Based Texture Analysis for Stratifying the Risk of Short-Term and Long-Term Breast Cancer in Screening

**DOI:** 10.3390/diagnostics14161823

**Published:** 2024-08-21

**Authors:** Bolette Mikela Vilmun, George Napolitano, Andreas Lauritzen, Elsebeth Lynge, Martin Lillholm, Michael Bachmann Nielsen, Ilse Vejborg

**Affiliations:** 1 Department of Diagnostic Radiology, Copenhagen University Hospital, Rigshospitalet, Blegdamsvej 9, 2100 Copenhagen, Denmark; 2 Department of Breast Examinations, Copenhagen University Hospital—Herlev and Gentofte, Gentofte Hospitalsvej 1, 2900 Hellerup, Denmark; 3 Department of Clinical Medicine, University of Copenhagen, Blegdamsvej 3B, 2200 Copenhagen, Denmark; 4 Department of Public Health, University of Copenhagen, Øster Farimagsgade 5, 1014 Copenhagen, Denmark; 5 Biomediq A/S, Strandlinien 59, 2791 Dragør, Denmark; 6 Nykøbing Falster Hospital, University of Copenhagen, Fjordvej 15, 4300 Nykøbing Falster, Denmark; 7 Department of Computer Science, University of Copenhagen, Universitetsparken 1, 2100 Copenhagen, Denmark

**Keywords:** breast cancer screening, mammography, breast density, parenchymal texture

## Abstract

Assessing a woman’s risk of breast cancer is important for personalized screening. Mammographic density is a strong risk factor for breast cancer, but parenchymal texture patterns offer additional information which cannot be captured by density. We aimed to combine BI-RADS density score 4th Edition and a deep-learning-based texture score to stratify women in screening and compare rates among the combinations. This retrospective study cohort study included 216,564 women from a Danish populations-based screening program. Baseline mammograms were evaluated using BI-RADS density scores (1–4) and a deep-learning texture risk model, with scores categorized into four quartiles (1–4). The incidence rate ratio (IRR) for screen-detected, interval, and long-term cancer were adjusted for age, year of screening and screening clinic. Compared with subgroup B1-T1, the highest IRR for screen-detected cancer were within the T4 category (3.44 (95% CI: 2.43–4.82)−4.57 (95% CI: 3.66–5.76)). IRR for interval cancer was highest in the BI-RADS 4 category (95% CI: 5.36 (1.77–13.45)−16.94 (95% CI: 9.93–30.15)). IRR for long-term cancer increased both with increasing BI-RADS and increasing texture reaching 5.15 (4.31–6.16) for the combination of B4-T4 compared with B1-T1. Deep-learning-based texture analysis combined with BI-RADS density categories can reveal subgroups with increased rates beyond what density alone can ascertain, suggesting the potential of combining texture and density to improve risk stratification in breast cancer screening.

## 1. Introduction

Mammographic density is an independent risk factor for breast cancer and is also known to mask malignancies [1,2]. Consequently, the sensitivity of mammography screening is lower, and the interval cancer rate is higher for women with dense versus non-dense breast tissue [3,4,5,6]. Women with high mammographic density have a higher risk of developing breast cancer, compared to those with low density [7]. Therefore, the current one-size-fits-all policy in mammography screening is not equitable [8]. Developing a more personalized screening program that considers breast density is discussed widely [9]. In the US, legislation mandates that physicians inform women of their mammographic density if categorized as heterogeneously dense or extremely dense, according to Breast Imaging-Reporting and Data system (BI-RADS) density score, to discuss the possibility of supplemental imaging. In 2022, the European Society of Breast Imaging began recommending supplemental screening for women with high density, and it is now also recommended by the European Commission Initiative on Breast Cancer [10,11]. However, studies have indicated that relying solely on mammographic density as the primary risk factor when tailoring screening strategies for women may not be sufficient [5,12]. 

Other than mammographic density, mammographically derived risk factors, such as assessment of parenchymal texture patterns, have been shown to be associated with a risk of breast cancer independently of density [13,14,15,16,17]. Deep learning, a subfield of artificial intelligence, uses multiple hidden layers of neural networks to process and analyze complex datasets [18]. This method provides an alternative to standard breast cancer risk models, potentially improving breast cancer prediction accuracy [19]. Previous studies have focused on the discriminatory accuracy, but their clinical utility and potential consequences in a clinical setting are rarely examined. Lauritzen et al. developed a deep-learning-based texture model for long-term breast cancer risk, which led to the identification of a subgroup of high-risk women from a screening cohort [20]. Breast density and parenchymal texture are correlated, but nevertheless, combining BI-RADS density score 4th Edition and a texture score, Euler-Chelpin et al. identified subgroups of women for whom the screening sensitivity was lower than indicated by the BI-RADS score alone [21]. 

Moving toward personalizing screening strategies, we should investigate the possibility of using different mammographic characteristics available at the screening site to better identify and understand the subgroups of women who could benefit from new screening schemes. 

The aim of this study was to investigate the clinical impact of stratifying women according to their different mammographic types of breast tissue, stratified into subgroups by combining BI-RADS density score 4th Edition and a deep-learning-based texture risk score, both assessed in a baseline screening mammogram in a population-based screening program. The objectives are to compare the rate of short-term breast cancer (STC) and long-term breast cancer (LTC) among combinations of BI-RADS density score and texture score. 

## 2. Materials and Methods

Our retrospective study was approved by the Danish Patient Safety Authority (3-3013-2961/1 and R-23025845. AD 2019/2021). 

### 2.1. Study Population

**Figure 1:**
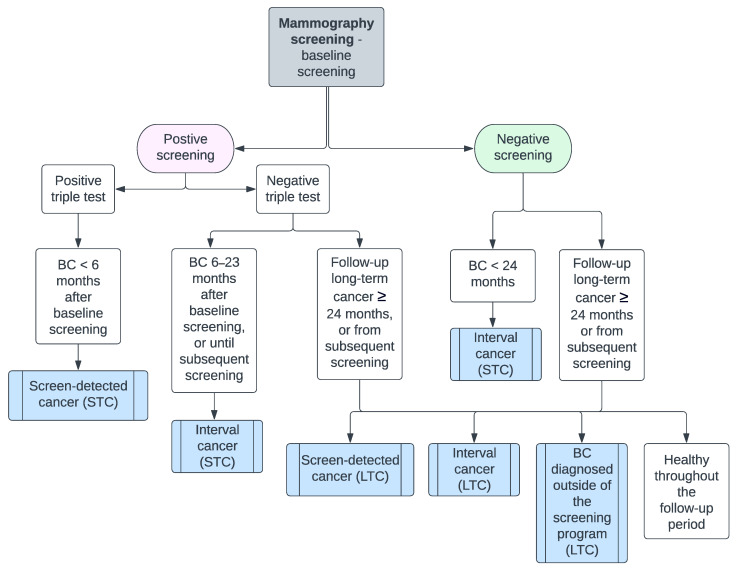
Flowchart, Capital Mammography Screening Program, Denmark, 2012–2023. BC = breast cancer, STC = short-term cancer, LTC = long-term cancer.

The Capital Region Mammography Screening Program in Denmark offers population-based biennial screening, free of charge, to all women aged 50–69 years at five public screening clinics. The study participants were women attending routine mammography screening between 1 November 2012, and 15 April 2021. A woman will enter the study at her first available mammography screening within the study period, available for texture scoring (referred to as the baseline screening). Women with breast cancer diagnosed before the baseline screening were excluded from the study. For screenings with suspicious findings (positive screening), diagnostic assessment was performed using the triple test, including clinical mammography, ultrasound, and biopsy. A cancer diagnosis was confirmed histologically. Women with a negative screening result were invited to be screened again in two years. Screen-detected cancer was defined as invasive carcinoma or ductal carcinoma in situ (DCIS) diagnosed within six months of a positive baseline screening. Interval cancer was defined as invasive carcinoma or DCIS diagnosed within 24 months after a negative baseline screening or 6–24 months after a positive baseline screening and negative diagnostic assessment, or before subsequent screening, whichever came first. Screen-detected and interval cancers were defined as STCs, see Figure 1. LTCs were defined as all breast cancers (invasive and DCIS) diagnosed more than 24 months after baseline screening, or diagnosed at a subsequent screening, whichever came first. Histological data were retrieved up until 15 April 2023, such that all women had at least 24 months of follow-up time.

For the analysis of LTCs, the study population was restricted to women without a STC diagnosis. In cases where a woman was diagnosed with both invasive carcinoma and DCIS, we classified the cancer as invasive carcinoma. Information on screening outcomes was retrieved by linkage based on unique personal identification numbers from the Radiology Information System (RIS), the Danish Civil Registration System, the Danish Breast Cancer Group, and the Danish Pathology Register. 

### 2.2. Image Technique and Interpretation

All women in the cohort received standard two-view (craniocaudal (CC) and mediolateral oblique (MLO)) FFDM. Women with subpectoral breast implants received additional views after pushback. Screening examinations were performed using Mammomat Inspiration or Revelation systems, Siemens AG, Germany. FFDM examinations were centrally and independently double read by two full-time breast radiologists. From November 2021 and forth AI was implemented as first reader in the low-risk group (stratified by AI). If the two readers disagreed on the need for recall, the final result was determined by consensus or arbitration with a third senior radiologist. At least one of the readers is a senior breast radiologist reading more than 3000 mammograms yearly. 

### 2.3. Breast Density

Breast density was coded using the 4th Edition BI-RADS density code [22] based on the CC and MLO projection. BI-RADS 1 is almost entirely fat (<25% fibroglandular tissue); BI-RADS 2 scattered 25–50% fibroglandular tissue; BI-RADS 3 heterogeneously (51–75%) dense; and BI-RADS 4 dense (>75%). The highest code was used if the two readers disagreed on the density code.

### 2.4. Deep-Learning-Based Texture Model

The mammographic texture deep-learning model was trained to estimate the long-term risk of breast cancer cross-vendor. In-depth methodological and technical details were previously reported by Lauritzen et al. [20]. The texture model was trained on a Dutch dataset consisting of 41,242 women with 1326 cancers sampled from the Dutch screening program in Utrecht, the Netherlands, from 1 January 2003, to 1 January 2015. The Dutch screening program offers biennial mammography screening to women between the ages of 50 and 74, using Hologic Lorad Selenia Systems. The model was trained to distinguish between women who remained healthy throughout the follow-up period of at least five years and those who developed breast cancer. Training images with a diagnosed cancer were excluded, so the model was trained on images of the cancer contra-lateral breast. The texture model therefore relies on features of healthy breast tissue to learn long-term risk. 

### 2.5. Statistical Analysis

We combined the BI-RADS categories with the texture categories to create 16 subgroups. The screen-detected cancer rate and interval cancer rate were calculated as the number of screen-detected or interval cancers per 1000 women within each subgroup. Incidence rates of SDCs, ICs, and LTCs were calculated as number of BC cases divided by person-years. For STCs, person-years were accumulated from baseline screening to BC diagnosis, 6 (for SDCs) or 24 (ICs) months after baseline, or subsequent screening, whichever came first. For LTCs, person-years were accumulated from the end date of STC follow-up period to BC diagnosis or 15 April 2023, whichever came first. Number of person-years in each group are presented in the Appendix A, Table A1. Incidence rates in different BI-RADS/texture subgroups were compared by computing incidence rate ratios (IRRs) and corresponding profile likelihood 95% confidence intervals (CIs), through Poisson regression models. For each outcome (SDC, IC, LTC), we fitted 3 different Poisson regression models with increasing number of covariate adjustments: Model 1 represents the crude model (no adjustment); Model 2 is adjusted for age at baseline, with age included in the model as a natural cubic spline with 3 degrees of freedom (this number was selected to minimize the Akaike Information Criterion among different choices of d.o.f. from 1 to 5); Model 3 is further adjusted for year of screening (categorical) and screening location (categorical: Bispebjerg Hospital, Bornholm Hospital, Herlev Hospital/Gentofte Hospital, Hillerød Hospital, Hvidovre Hospital). Furthermore, mixed effect models with random intercept were run to investigate the possible clustering effect of the screening locations, finding however no variability among them (data not reported). Number of women in each age category are presented in the Appendix Table A2. In the following, we only reported results from Model 3, whereas results from Model 1 and 2 can be found in the Appendix Table A3. All analyses and plots were made using R version 4.3.2 with package tidyverse [23].

## 3. Results

**Figure A1:**
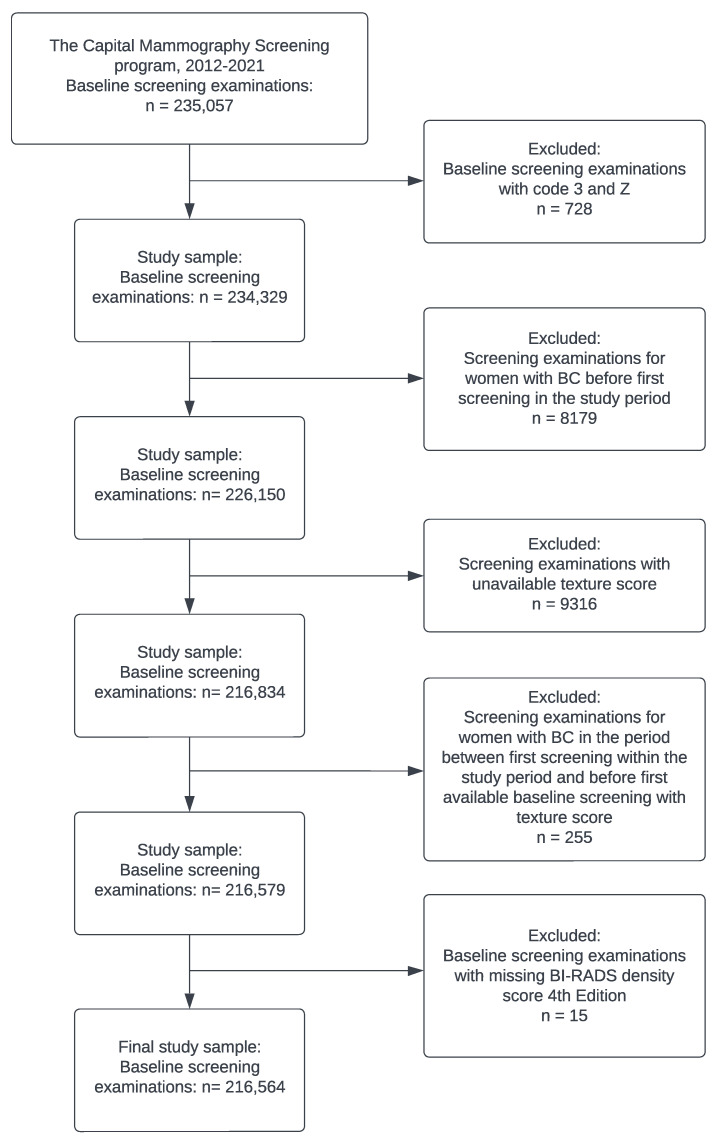
Flowchart describing sample selection, the Capital Mammography Screening Program, Denmark, 2012–2021. Code 3 refers to “Technical recall”. Code Z is when there is no examination. BC = Breast Cancer.

A total sample of 216,564 women with baseline screening examinations were included in the study (see Figure A1 for flowchart). The characteristics of the study sample are presented in Table 1. The median age of participants was 56.2 years (Q1–Q3: 51.7–63.0 years). A total of 2035 women were diagnosed with STC; 1485 screen-detected cancers, and 550 interval cancers (174 detected within the first year). Among the cohort of women analyzed for STC, 67,671 (31.2%) were classified as BI-RADS 1, 81,759 (37.8%) as BI-RADS 2, 54,918 (25.4%) as BI-RADS 3, and 12,216 (5.6%) as BI-RADS 4. There were 54,141 (25%) women in each texture category (number of women in each subgroup is presented in Table A4). In the analysis for LTC, women diagnosed with an STC were excluded, resulting in a total sample of 214,501 women with baseline screening examinations. Out of those, 67,275 (31.4%) women were classified as BI-RADS 1, 80,894 (37.7%) as BI-RADS 2, 54,285 (25.3%) as BI-RADS 3, and 12,047 (5.6%) as BI-RADS 4. There were 53,928 (25.1%) women in the T1, 53,748 (25.1%) in T2, 53,602 (25%) in T3, and 53,223 (24.8%) in T4. In total, 4687 women with long-term cancers were identified with a median time to diagnosis of 56.3 months (Q1–Q3: 36.3–80.2 months), Table A5. Overall, 1,158,577 person-years were accumulated for the analysis, Table A1.

### 3.1. Short-Term Cancer

The screen-detected cancer rate ranged from 1.63 to 13.68 per 1000 women, with the highest rate observed in the B2 + T4, see Table 2. The T4 subgroups consistently showed the highest rates ranging from 9.36 to 13.68 per 1000 women, whereas the lowest rates were observed mainly in B4 subgroups. Within each BI-RADS category, the screen-detected cancer rate increased by increasing level of texture.

The interval cancer rate ranged from 0.54 to 8.4 per 1000 women across the subgroups, increasing with increasing BI-RADS and texture score, Table 3. The lowest interval cancer rate was observed in B1 + T1 (0.54 per 1000 women), while the highest was in B4 + T4 (8.4 per 1000 women). The highest rates occurred in the BI-RADS 4 subgroups, ranging from 2.71 to 8.4 per 1000 women. 

**Figure 2:**
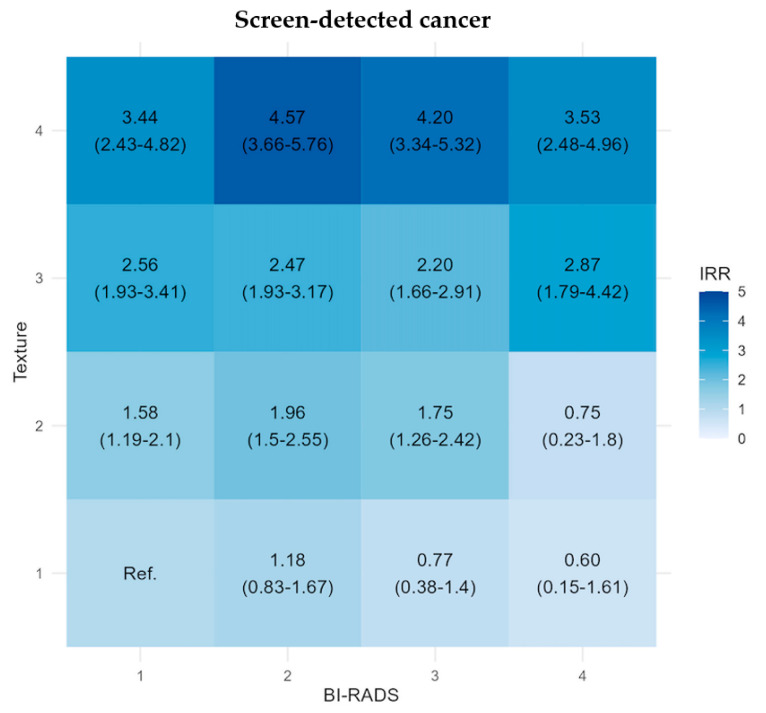
Matrix plots of the adjusted incidence rate ratio (IRR) with 95% confidence intervals for screen-detected cancers stratified by BI-RADS density score and texture score, the Capital Mammography Screening Program, Denmark, 2012–2023. IRR is adjusted by age, year of screening, and hospital of screening (model 3).

**Figure 3:**
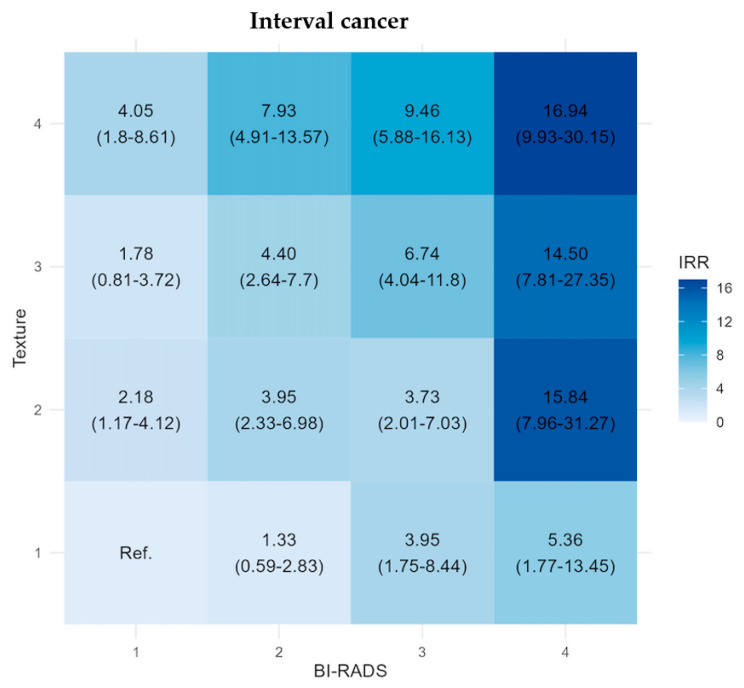
Matrix plots of the adjusted incidence rate ratio (IRR) with 95% confidence intervals for interval cancers stratified by BI-RADS density score and texture score, the Capital Mammography Screening Program, Denmark, 2012–2023. IRR is adjusted by age, year of screening, and hospital of screening (model 3).

Our main results from the regression Model 3 are reported in Figure 2 and Figure 3. The adjusted IRR for screen-detected cancer was highest in the T4 subgroups across the BI-RADS categories, ranging from 3.44 (95% CI: 2.43–4.82) to 4.57 (95% CI: 3.66–5.76), which were statistically significantly higher than the reference group, see Figure 2. The three subgroups with the lowest IRR (0.60 (95% CI: 0.15–1.61)–0.77 (95% CI: 0.38–1.4)) were observed in the B3 and B4 subgroups with low texture (non-significantly compared with the reference group).

The adjusted IRR for interval cancer shows a different pattern than for SDC, with increasing risk with increasing BI-RADS and texture. Notably, the highest IRR for IC (14.50 (95% CI: 7.81–27.35)–16.94 (95% CI: 9.93–30.15)) were observed in the top three subgroups all in the B4 category, indicating a distinct risk within these subgroups (statistically significantly higher than the reference group). Conversely, the three lowest IRR were observed in B1 and B2 categories (1.33 (95% CI: 0.59–2.83)–2.18 (95% CI: 1.17–4.12)). The rate in the B3 + T1 subgroup was similar to that of B1 + T4, showing the potential of considering both texture and BI-RADS categories when stratifying for breast cancer risk.

### 3.2. Long-Term Cancer

**Figure 4:**
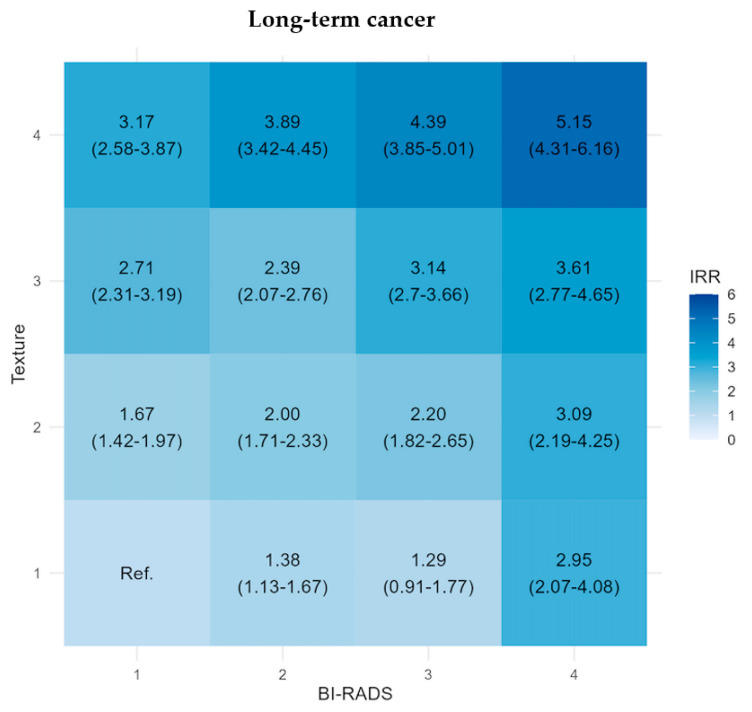
Matrix plots of the adjusted incidence rate ratio (IRR) with 95% confidence intervals for long-term cancer stratified by BI-RADS density score and texture score, Capital Mammography Screening Program, Denmark, 2012–2023. IRR is adjusted by age, year of screening, and hospital of screening.

The adjusted IRR increased for LTC with increasing BI-RADS and texture, with the subgroups exhibiting the highest IRR mainly being in the T4 and B4 subgroups (2.95 (95% CI: 2.07–4.08))–5.15 (95% CI: 4.31–6.16), Figure 4. When comparing the subgroups with the reference group B1 + T1, all subgroups except B3 + T1, showed statistically significantly increased IRR. Selecting subgroups with a threshold of IRR at 4.0 and above (B3 + T4 and B4 + T4), meaning a group of women with more than four times higher rate of LTC compared to the B1 + T1 group, comprise 12.48% of our screening cohort.

## 4. Discussion

In our study, we combined BI-RADS density score with a deep-learning-based texture score to risk stratify women aged between 50–69 at their baseline screening in a populations-based screening program. Our findings showed that texture enhanced breast cancer risk assessment beyond density alone, for both short-term and long-term risk. While one might anticipate the highest risk to be in BI-RADS 4, the use of texture revealed additional subgroups of high risk within lower density categories. Additionally, we identified subgroups within BI-RADS 3 and 4 categories combined with low texture scores, exhibiting a lower rate of breast cancer. 

Many studies have consistently demonstrated the significance of breast density as an independent risk factor and the potential to improve risk assessment and mammographic density is the leading measure for risk assessment [2,24,25]. The increased attention informing women of their breast density aims to highlight the risk associated with high density and the potential benefit of additional screening [26]. However, there is growing evidence that texture not only reflects the inherent biological risk for cancer development, but also parenchymal changes associated with breast cancer development [12,27]. A systematic review has shown that the addition of texture features to risk prediction models improves model performance beyond density [28], highlighting texture’s contribution to risk assessment [12,13,17,28,29]. Moreover, studies have shown texture to be able to distinguish BRCA1 and BRCA2 carriers from women of low risk of breast cancer, which is not the case for density [30]. 

Using a deep-learning-based texture model enables capturing important mammographic features associated with risk, instead of feeding the model prior assumptions [31]. Lauritzen et al. [20] developed and validated the deep-learning texture model used in this study, yielding a moderate AUC for interval cancers of 0.69/0.70 and 0.64 for LTC’s in their study, showing the potential of deep-learning texture for risk assessment for both short- and long-term breast cancer risk. Additionally, a systematic review by Schopf et al. showed that AI image-only risk models yielded a median AUC of 0.72 (0.61–0.90), outperforming model performance of density only or clinical risk factors (0.61 (0.54–0.69)) [32]. Using automatic mammographic biomarkers can facilitate swift risk assessment at the screening site.

Identifying women for whom mammography will not provide adequate detection of breast cancer and with a high risk of being diagnosed with interval cancer in the near future could shorten the time to diagnosis, enabling a more efficient screening program through tailored screening. Increased breast density is associated with masking, as well as risk, complicating cancer detection in women with high density. Both density and texture have more impact on interval cancers, than on the screen-detected and LTC’s. We found that the lowest IRR for screen-detected cancer, and the highest IRR for interval cancer, were within the BI-RADS 4 category, probably reflecting the masking potential of high density, especially for BI-RADS category 4. In the BI-RADS 2–3 categories combined with high texture, the difference between the risk of a screen-detected and interval cancer was more balanced. However, studies have shown that masking is not limited to higher BI-RADS categories, and that textural features can predict masking beyond density [33,34,35]. Short-term risk prediction is useful for guiding tailored screening strategies. The IRR for interval cancer in the B3 + T1 subgroup was similar to that of B1 + T4, showing the diverse risk patterns across the BI-RADS categories, see Figure 3. Identifying women at their baseline screening according to the risk of interval cancer and masking, enables the possibility of offering them a shorter screening interval or supplemental screening modalities, including digital breast tomosynthesis, ultrasound, or MRI, targeting masking and potentially increasing cancer detection in a screening program [8,36].

Long-term risk assessment can guide primary prevention efforts, in addition to tailoring screening strategies acknowledging that masking diminishes its impact on long-term breast cancer risk [17,37,38]. Selecting women with BI-RADS density 3 and 4 for supplemental screening would in our study encompass approximately 150,000 women or 35% of our cohort, with an IRR for LTC ranging from 1.29 (95% CI: 0.91–1.77) to 5.15 (95% CI: 4.31–6.16) through the subgroups. This would impose a considerable burden on a screening program and relying solely on BI-RADS density would lead to unnecessary or shortened screening intervals for low-risk, high density women, whereas women with low-density and high-risk would miss out on relevant screening [5]. Our results support further stratification beyond density, showing the highest rates for LTC where found primarily in the subgroups of the highest texture quartile. 

This study has some limitations, including the small size of some subgroups, limiting the reliability of our estimates. Additionally, the use of baseline screening may not accurately represent the true baseline screening for all women. Lack of data on immigration status or death could introduce bias due to loss of follow-up. However, the strength of our study was the large cohort from a real-life population-based screening program with long follow-up. Furthermore, the deep-learning-based texture model was trained cross-vendor, improving the reliability of our findings across different vendors. 

## 5. Conclusions

Our results have shown a broad range of cancer detection across subgroups, emphasizing the importance of tailored screening strategies. The rates for screen-detected cancer were highest in the T4 subgroups, whereas the lowest rates were observed in the B4 subgroups. For interval cancer, the highest rates were observed in the three B4 + T2-4 subgroups. When analyzing LTC, the highest rates were observed in the T4 and B4 subgroups combined. In our study, high density does not necessarily indicate a high rate of breast cancer, and conversely, subgroups of women with low density does not equate to a low rate of breast cancer. Using deep-learning-based texture analysis within BI-RADS density categories improves risk stratification in breast cancer screening beyond density alone. Further research is still warranted to guide screening strategies tailored to individual breast cancer risk profiles.

## Figures and Tables

**Table 1:** Cohort characteristics, the Capital Region Mammography Screening Program, Denmark, 2012–2023. DCIS = Ductal carcinoma in situ, IC = interval cancer, LTC = long-term cancer, SDC = screen-detected cancer.

	Women at Risk of Short-Term Cancer (Invasive + DCIS)	Women at Risk of Long-Term Cancer (Invasive + DCIS)
No. of women with baseline screenings	216,564	214,501
Median age, years (Q1–Q3)	56.2 (51.7–63.0)	56.1 (51.7–63.0)
SDC, n	1485	-
Of which invasive	1226	-
Of which DCIS	259	-
IC, n	550	-
LTC, n	-	4687
BI-RADS 1, no. of women (%)	67,671 (31.2%)	67,275 (31.4%)
BI-RADS 2, no. of women (%)	81,759 (37.8%)	80,894 (37.7%)
BI-RADS 3, no. of women (%)	54,918 (25.4%)	54,285 (25.3%)
BI-RADS 4, no. of women (%)	12,216 (5.6%)	12,047 (5.6%)
Texture 1, no. of women (%) (0.000696–0.0513)	54,141 (25%)	53,928 (25.1%)
Texture 2, no. of women (%) (0.0513–0.0925)	54,141 (25%)	53,748 (25.1%)
Texture 3, no. of women (%) (0.0925–0.157)	54,141 (25%)	53,602 (25%)
Texture 4, no. of women (%) (0.157–0.929)	54,141 (25%)	53,223 (24.8%)

**Table 2:** Screen-detected cancer rate in subgroups stratified by combining BI-RADS density score and texture score. The Capital Mammography Screening Program, Denmark, 2012–2023. B = BI-RADS density score 4th edition corresponding to each of the four categories, T = texture quartile corresponding to each of the four quartiles.

Screen-Detected Cancer Rate Per 1000 Women	BI-RADS 1	BI-RADS 2	BI-RADS 3	BI-RADS 4	Any BI-RADS
Texture 1	2.99	3.33	2.09	1.63	4.88
Texture 2	4.87	5.66	4.83	2.01	7.94
Texture 3	8.07	7.36	6.12	7.62	7.76
Texture 4	10.8	13.68	11.78	9.36	6.55
Any Texture	2.96	5.08	7.15	12.25	6.86

**Table 3:** Interval cancer rate in subgroups stratified by combining BI-RADS density score and texture score. The Capital Mammography Screening Program, Denmark, 2012–2023. B = BI-RADS density score 4th edition corresponding to each of the four categories, T = texture quartile corresponding to each of the four quartiles.

Interval Cancer Rate Per 1000 Women	BI-RADS 1	BI-RADS 2	BI-RADS 3	BI-RADS 4	Any BI-RADS
Texture 1	0.54	0.71	2.09	2.71	0.9
Texture 2	1.19	2.1	1.95	8.04	2.51
Texture 3	0.98	2.35	3.49	7.31	3.57
Texture 4	2.21	4.2	4.82	8.4	7.2
Any Texture	0.79	1.98	2.7	4.69	2.54

## Data Availability

The data presented in this study are available on reasonable request. The data are not publicly available due to privacy restrictions.

## Appendix A

Clinical significance of combined density and deep-learning-based texture analysis for stratifying the risk of short-term and long-term breast cancer in screening.

**Table A1:** Person-years and rate per 1000 person-years for short-term cancers (STC) stratified by combining BI-RADS density score, deep-learning-based texture score, and by combination, the Capital Region Mammography Screening Program, Denmark, 2012–2023. IC = interval cancer, SDC = screen-detected cancer. B = BI-RADS density score 4th edition corresponding to each of the four categories, T = texture quartile corresponding to each of the four quartiles.

	Number of Women with Baseline Screening n (%)	Person-Years SDC	SDC Rate Per 1000 Person-Years	Person-Years IC	IC Rate Per 1000 Person-Years	Person-Years LTC	LTC Rate Per 1000 Person-Years
**Total**	216,564 (100%)	106,105.3	14	424,558.94	1.3	1,158,577	4.05
**B1 + T1**	33,392 (15.4%)	16,413.47	6.09	65,708.38	0.27	186,106	1.63
**B1 + T2**	18,469 (8.5%)	9064.02	9.93	362,573	0.61	102,546	2.76
**B1 + T3**	11,275 (5.2%)	5518.79	16.49	22,059.63	0.5	63,114	4.52
**B1 + T4**	4535 (2.1%)	2214.66	22.13	8843.84	1.13	25,763	5.28
**B2 + T1**	14,118 (6.5%)	6937.89	6.77	27,796.89	0.36	67,106	2.16
**B2 + T2**	21,892 (10.1%)	10,737.66	11.55	42989	1.07	112,563	3.18
**B2 + T3**	23,374 (10.8%)	11,447.95	15.02	45,782.36	1.2	129,549	3.87
**B2 + T4**	22,375 (10.3%)	10,898.48	28.08	43,531.16	2.16	132,586	6.32
**B3 + T1**	4789 (2.2%)	2356	4.24	9442.34	1.06	19,923	1.96
**B3 + T2**	11,791 (5.4%)	5787.83	9.85	23,205.86	0.99	52,183	3.35
**B3 + T3**	16,344 (7.5%)	8012.83	12.48	32,090.63	1.78	79,078	4.83
**B3 + T4**	21,994 (10.2%)	10,729.64	24.14	42,920.38	2.47	130,195	6.81
**B4 + T1**	1842 (0.9%)	906.02	3.31	3633.15	1.38	8591	4.42
**B4 + T2**	1989 (0.9%)	977.77	4.09	3911.26	4.09	8790	4.55
**B4 + T3**	3148 (1.5%)	1541.7	15.57	6162.52	3.73	13,600	5.29
**B4 + T4**	5237 (2.4%)	2560.6	19.14	10,224.23	4.3	26,884	7.59
**BI-RADS density and Texture categories**							
**B1**	67,671 (31.2%)	33,210.93	9.94	132,869.14	0.46	377,529	2.67
**B2**	81,759 (37.8%)	40,021.98	16.22	160,099.42	1.28	441,803	4.17
**B3**	54,918 (25.4%)	26,886.29	15.84	107,659.21	1.82	281,379	5.27
**B4**	12,216 (5.6%)	5986.09	13.36	23,931.16	3.68	57,865	6.12
**T1**	54,141 (25%)	26,613.39	6.01	106,580.77	0.4	281,726	1.87
**T2**	54,141 (25%)	26,567.27	10.35	106,363.41	1.01	276,083	3.1
**T3**	54,141 (25%)	26,521.28	14.59	106,095.15	1.38	285,341	4.35
**T4**	54,141 (25%)	26,403.37	25.11	105,519.61	2.41	315,427	6.55

**Table A2:** Number of women included in this study, stratified by BI-RADS density score and deep-learning-based texture score, and by combination, and further stratified by age. The Capital Region Mammography Screening Program, Denmark, 2012–2023. B = BI-RADS density score 4th edition corresponding to each of the four categories, T = texture quartile corresponding to each of the four quartiles.

	50–55	55–60	60–65	65-	Total
**Total**	98,516	41,413	36,985	39,650	216,564
**B1 + T1**	12,412	7321	6594	7065	33,392
**B1 + T2**	6317	3717	3916	4519	18,469
**B1 + T3**	3394	2115	2485	3281	11,275
**B1 + T4**	1312	783	1042	1398	4535
**B2 + T1**	7874	2718	1948	1578	14,118
**B2 + T2**	10,519	4331	3585	3457	21,892
**B2 + T3**	9310	4849	4385	4830	23,374
**B2 + T4**	7494	4583	4710	5588	22,375
**B3 + T1**	3297	699	433	360	4789
**B3 + T2**	7787	1789	1182	1033	11,791
**B3 + T3**	9871	2713	2008	1752	16,344
**B3 + T4**	10,377	4190	3663	3764	21,994
**B4 + T1**	1227	278	177	160	1842
**B4 + T2**	1476	222	148	143	1989
**B4 + T3**	2349	374	205	220	3148
**B4 + T4**	3500	731	504	502	5237
**BI-RADS density and Texture categories**					
**B1**	23,435	13,936	14,037	16,263	67,671
**B2**	35,197	16,481	14,628	15,453	81,759
**B3**	31,332	9391	7286	6909	54,918
**B4**	8552	1605	1034	1025	12,216
**T1**	24,810	11,016	9152	9163	54,141
**T2**	26,099	10,059	8831	9152	54,141
**T3**	24,924	10,051	9083	10,083	54,141
**T4**	22,683	10,287	9919	11,252	54,141

**Table A3:** Model 1 and 2 for screen-detected, interval and long-term cancer. Model 1 represents the crude model (no adjustment); Model 2 is adjusted for age at baseline, with age included in the model as a natural cubic spline with 3 degrees of freedom (this number was selected to minimize the Akaike Information Criterion among different choices of d.o.f. from 1 to 5).

	Screen-Detected Cancer		Interval Cancer		Long-Term Cancer	
	Model 1 (95% CI)	Model 2 (95% CI)	Model 1 (95% CI)	Model 2 (95% CI)	Model 1 (95% CI)	Model 2 (95% CI)
**B1 + T1**	Ref (1.00)	Ref (1.00)	Ref (1.00)	Ref (1.00)	Ref (1.00)	Ref (1.00)
**B1 + T2**	1.63 (1.22–2.17)	1.58 (1.19–2.1)	2.22 (1.19–4.18)	2.19 (1.18–4.14)	1.69 (1.44–1.99)	1.68 (1.43–1.97)
**B1 + T3**	2.71 (2.04–3.59)	2.54 (1.91–3.38)	1.82 (0.83–3.8)	1.78 (0.81–3.71)	2.76 (2.35–3.25)	2.71 (2.31–3.19)
**B1 + T4**	3.63 (2.56–5.08)	3.37 (2.37–4.71)	4.13 (1.83–8.77)	4.02 (1.78–8.54)	3.23 (2.63–3.95)	3.16 (2.57–3.86)
**B2 + T1**	1.11 (0.78–1.56)	1.21 (0.85–1.71)	1.31 (0.58–2.79)	1.37 (0.61–2.92)	1.32 (1.08–1.61)	1.39 (1.14–1.69)
**B2 + T2**	1.9 (1.46–2.47)	1.98 (1.52–2.56)	3.91 (2.31–6.91)	4 (2.36–7.08)	1.95 (1.67–2.27)	2 (1.72–2.34)
**B2 + T3**	2.47 (1.93–3.16)	2.47 (1.94–3.17)	4.39 (2.63–7.68)	4.41 (2.64–7.71)	2.37 (2.05–2.73)	2.39 (2.07–2.75)
**B2 + T4**	4.61 (3.69–5.8)	4.46 (3.57–5.62)	7.88 (4.89–13.48)	7.79 (4.83–13.32)	3.87 (3.4–4.42)	3.84 (3.37–4.39)
**B3 + T1**	0.7 (0.34–1.27)	0.79 (0.39–1.44)	3.87 (1.72–8.22)	3.87 (1.72–8.22)	1.2 (0.85–1.65)	1.31 (0.93–1.81)
**B3 + T2**	1.62 (1.16–2.23)	1.81 (1.3–2.5)	3.61 (1.96–6.79)	3.85 (2.08–7.24)	2.05 (1.7–2.47)	2.24 (1.85–2.69)
**B3 + T3**	2.05 (1.55–2.7)	2.24 (1.7–2.96)	6.48 (3.9–11.33)	6.82 (4.09–11.92)	2.96 (2.54–3.44)	3.16 (2.57–3.86)
**B3 + T4**	3.96 (3.16–5.01)	4.1 (3.27–5.19)	9.02 (5.62–15.35)	9.2 (5.73–15.67)	4.17 (3.67–4.76)	4.32 (3.79–4.92)
**B4 + T1**	0.54 (0.13–1.44)	0.61 (0.15–1.62)	5.02 (1.66–12.59)	5.35 (1.77–13.43)	2.71 (1.9–3.74)	2.71 (1.9–3.74)
**B4 + T2**	0.67 (0.21–1.6)	0.76 (0.23–1.81)	14.93 (7.53–29.34)	16.13 (8.11–31.78)	2.79 (1.97–3.82)	3.12 (2.21–4.29)
**B4 + T3**	2.56 (1.6–3.92)	2.91 (1.82–4.47)	13.62 (7.37–25.57)	14.74 (7.95–27.76)	3.24 (2.49–4.16)	3.65 (2.8–4.7)
**B4 + T4**	3.4 (2.21–4.39)	3.49 (2.45–4.89)	15.71 (9.24–27.87)	16.71 (9.81–29.71)	4.65 (3.89–5.54)	5.11 (4.27–6.11)

**Table A4:** Characteristics for short-term cancer (STC) stratified by BI-RADS density score, deep-learning-based texture score, and by combination of BI-RADS density score and deep-learning-based texture score, the Capital Region Mammography Screening Program, Denmark, 2012–2023. B = BI-RADS density score 4th edition corresponding to each of the four categories, T = texture quartile corresponding to each of the four quartiles.

	Number of Women with Baseline Screening n (%)	All STC, n	Screen-Detected Cancer, n (%)	Interval Cancer, n (%)
**Total**	216,564 (100%)	2035	1485 (100%)	550 (100%)
**B1 + T1**	33,392 (15.4%)	118	100 (6.7%)	18 (3.3%)
**B1 + T2**	18,469 (8.5%)	112	90 (6.6%)	22 (4%)
**B1 + T3**	11,275 (5.2%)	102	91 (6.1%)	11 (2%)
**B1 + T4**	4535 (2.1%)	59	49 (3.3%)	10 (1.8%)
**B2 + T1**	14,118 (6.5%)	57	47 (3.2%)	10 (1.8%)
**B2 + T2**	21,892 (10.1%)	170	124 (8.4%)	46 (8.4%)
**B2 + T3**	23,374 (10.8%)	227	172 (11.6%)	55 (10%)
**B2 + T4**	22,375 (10.3%)	400	306 (20.6%)	94 (17.1%)
**B3 + T1**	4789 (2.2%)	20	10 (0.7%)	10 (1.8%)
**B3 + T2**	11,791 (5.4%)	80	57 (3.8%)	23 (4.2%)
**B3 + T3**	16,344 (7.5%)	157	100 (6.7%)	57 (10.4%)
**B3 + T4**	21,994 (10.2%)	365	259 (17.4%)	106 (19.3%)
**B4 + T1**	1842 (0.9%)	8	3 (0.2%)	5 (0.9%)
**B4 + T2**	1989 (0.9%)	20	4 (0.3%)	16 (2.9%)
**B4 + T3**	3148 (1.5%)	47	24 (1.6%)	23 (4.2%)
**B4 + T4**	5237 (2.4%)	93	49 (3.3%)	44 (8%)
**BI-RADS density and Texture categories**				
**B1**	67,671 (31.2%)	391	330 (22.2%)	61 (11.1%)
**B2**	81,759 (37.8%)	854	649 (43.7%)	205 (37.3%)
**B3**	54,918 (25.4%)	622	426 (28.7%	196 (35.6%)
**B4**	12,216 (5.6%)	168	80 (5.4%)	88 (16%)
**T1**	54,141 (25%)	203	160 (10.8%)	43 (7.8%)
**T2**	54,141 (25%)	382	275 (18.5%)	107 (19.5%)
**T3**	54,141 (25%)	533	387 (26.1%)	146 (26.6%)
**T4**	54,141 (25%)	917	663 (44.7%)	254 (46.2%)

**Table A5:** Characteristics for long-term cancer (LTC) stratified by BI-RADS density score, deep-learning-based texture score, and by combination of BI-RADS density score and deep-learning-based texture score, the Capital Region Mammography Screening Program, Denmark, 2012–2023. DCIS = ductal carcinoma in situ, Q1–3 = interquartile 1–3, B = BI-RADS density score 4th edition corresponding to each of the four categories, T = texture quartile corresponding to each of the four quartiles.

	Number of Women with Baseline Screening, n (%)	LTC, n	LTC, of Which Invasive Carcinoma, n	LTC, of Which DCIS, n	Median Time to LTC, Counted from Baseline, Months (Q1–Q3)
**Total**	214,501 (100%)	4687 (100%)	4088	599	56.3 (36.3–80.2)
**B1 + T1**	33,271 (15.5%)	304 (6.4%)	276	28	52.8 (34.2–78.3)
**B1 + T2**	18,357 (8.6%)	283 (6%)	255	28	54.1 (32.7–78.3)
**B1 + T3**	11,171 (5.2%)	285 (6.1%)	252	33	53.4 (35.6–80.3)
**B1 + T4**	4476 (2.1%)	136 (2.9%)	120	16	52.9 (31.9–79)
**B2 + T1**	14,056 (6.6%)	145 (3.1%)	125	20	61.8 (34.2–83.2)
**B2 + T2**	21,717 (10.1%)	358 (7.6%)	309	49	56 (35.8–79.3)
**B2 + T3**	23,146 (10.8%)	501 (10.7%)	439	62	54.8 (34.5–81.1)
**B2 + T4**	21,975 (10.2%)	838 (17.9%)	746	92	58.1 (38.7–82.8)
**B3 + T1**	4767 (2.2%)	39 (0.8%)	30	9	60.3 (32.2–82.6)
**B3 + T2**	11,706 (5.5%)	175 (3.7%)	141	34	51.6 (31.4–75.3)
**B3 + T3**	16,184 (7.5%)	382 (8.2%)	319	63	53.5 (33.5–77.4)
**B3 + T4**	21,628 (10.1%)	887 (18.9%)	777	110	60.6 (44.1–83.6)
**B4 + T1**	1834 (0.9%)	38 (0.8%)	34	4	57 (42.4–78)
**B4 + T2**	1968 (0.9%)	40 (0.9%)	31	9	57.5 (38.2–75.5)
**B4 + T3**	3101 (1.4%)	72 (1.5%)	58	14	54.5 (32–78.3)
**B4 + T4**	5144 (2.5%)	204 (4.4%)	176	28	58.8 (41–79.8)
**BI-RADS density and Texture categories**					
**B1**	67,275 (31.4%)	1008 (21.5%)	903	105	53 (34.2–78.6)
**B2**	80,894 (37.7%)	1842 (39.3%)	1619	223	57.3 (36.1–81.9)
**B3**	54,285 (25.3%)	1483 (31.6%)	1267	216	57.5 (36.8–80.4)
**B4**	12,047 (5.6%)	354 (7.6%)	299	55	57.1 (39.1–78.6)
**T1**	53,928 (25.1%)	526 (11.2%)	465	61	55.6 (34.3–80.1)
**T2**	53,748 (25.1%)	856 (18.3%)	736	120	53.3 (34–78.2)
**T3**	53,602 (25%)	1240 (26.5%)	1068	172	54.1 (34.2–78.9)
**T4**	53,223 (24.8%)	2065 (44.1%)	1819	246	59.4 (40.6–82.7)
